# A patch-based bolus technique for total scalp irradiation: dosimetric impact of conformity and reproducibility

**DOI:** 10.1093/jrr/rrag069

**Published:** 2026-08-13

**Authors:** Takahiro Kato, Hiroshi Niwayama, Yuki Tominaga, Yosuke Nagasawa, Daiki Senzaki, Takaomi Harada, Hisashi Sato

**Affiliations:** Department of Radiological Sciences, Graduate School of Health Sciences, Fukushima Medical University, 10-6 Sakaemachi, Fukushima, 960-8516, Japan; Department of Radiology, Ohta Nishinouchi Hospital, 2-5-20 Nishinouchi, Koriyama, Fukushima, 963-8558, Japan; Graduate School of Biomedical Sciences, Tokushima University, 2-24 Shinkura-cho, Tokushima, 770-8501, Japan; Medical Physics Laboratory, Division of Health Science, Graduate School of Medicine, The University of Osaka, 1-7 Yamadaoka, Suita, Osaka, 565-0871, Japan; Department of Radiological Sciences, Graduate School of Health Sciences, Fukushima Medical University, 10-6 Sakaemachi, Fukushima, 960-8516, Japan; Department of Radiology, Fukushima Medical University Hospital, 1 Hikarigaoka, Fukushima, 960-1295, Japan; Department of Radiological Sciences, Graduate School of Health Sciences, Fukushima Medical University, 10-6 Sakaemachi, Fukushima, 960-8516, Japan; Department of Radiological Sciences, Graduate School of Health Sciences, Fukushima Medical University, 10-6 Sakaemachi, Fukushima, 960-8516, Japan; Department of Radiological Sciences, Graduate School of Health Sciences, Fukushima Medical University, 10-6 Sakaemachi, Fukushima, 960-8516, Japan

**Keywords:** bolus, HyperArc, scalp angiosarcoma, total scalp irradiation, noncoplanar

## Abstract

Total scalp irradiation (TSI) for scalp angiosarcoma remains technically challenging because of the large, highly curved target geometry and the need for reliable bolus application. This study aimed to develop a practical method to improve bolus conformity using commercially available bolus and to evaluate the dosimetric impact of bolus reproducibility in volumetric-modulated arc therapy (VMAT)–based TSI. An anthropomorphic head phantom was used to simulate TSI conditions. A three-segment ‘patch bolus’ method was developed to enhance conformity and setup stability. Treatment plans were generated using HyperArc with identical optimization parameters for three conditions: virtual bolus, single-sheet bolus and patch bolus. To assess reproducibility, the patch bolus was reapplied and rescanned 10 times, and dose distributions were recalculated on each dataset after rigid registration to isolate bolus-related variations. The patch bolus method substantially reduced air gaps compared with the single-sheet approach and achieved target coverage comparable to the virtual bolus condition. In contrast, the single-sheet bolus resulted in inferior coverage in regions with poor contact. Repeated application of the patch bolus demonstrated high reproducibility, with variations within 2% for most dose–volume metrics. Although relative variations exceeding 5% were observed for the lens, absolute doses remained low and clinically negligible. These findings indicate that VMAT-based TSI is relatively robust against minor bolus variations. The proposed patch bolus method provides improved conformity, reproducibility and workflow efficiency, representing a simple and clinically feasible strategy for TSI using external beam radiotherapy.

## INTRODUCTION

Scalp angiosarcoma, a rare malignant neoplasm of endothelial origin arising from the skin, is characterized by an extremely aggressive clinical course [1, 2]. The disease has a high propensity for early local recurrence and distant metastasis, most commonly to the lungs. It predominantly affects older patients, and at the time of diagnosis, the lesions often extend diffusely over the entire scalp. Although surgical resection remains the cornerstone of treatment, complete curative excision is frequently unfeasible because of extensive tumor spread, advanced age, poor general condition or cosmetic considerations. Consequently, radiotherapy plays a pivotal role in the management of unresectable disease and as adjuvant treatment following surgery [3, 4].

In radiotherapy for scalp angiosarcoma, high total doses of ~70 Gy are commonly prescribed to achieve improved local control, and total scalp irradiation (TSI) is therefore widely used. However, TSI is technically challenging because the target volume is large, geometrically complex and helmet-shaped, located immediately beneath the skin surface and in proximity to critical organs such as the brain, lenses and optic nerves. Achieving optimal dose distribution is therefore particularly demanding. Notably, even low-dose irradiation of normal brain tissue has been associated with late neurocognitive impairment [5, 6], making the minimization of brain dose while maintaining adequate target coverage a major clinical concern.

External beam radiotherapy using photons or electrons has been widely adopted for TSI [7, 8]; however, both modalities require the use of bolus materials to ensure adequate dose delivery to superficially located tumors. In recent years, advanced photon techniques such as intensity-modulated radiotherapy (IMRT), volumetric-modulated arc therapy (VMAT) and helical tomotherapy, have been explored for this indication [4, 9–11]. Despite their superior dose conformity, the impact of commercially available bolus conformity and setup reproducibility on dose distribution in these high-precision techniques has not been fully characterized and remains an important issue to be addressed. Achieving consistent and accurate bolus contact over the entire scalp is inherently challenging, and few studies have systematically investigated strategies to improve the fitting accuracy of commercially available bolus materials. Therefore, the purpose of this study was to investigate a practical method to enhance bolus conformity using commercially available bolus and to experimentally evaluate the dosimetric impact of bolus reproducibility in VMAT-based TSI.

## MATERIALS AND METHODS

### Contouring and bolus application

An adult head phantom (PBU-60; Kyoto Kagaku, Kyoto, Japan) was used in this study. Computed tomography (CT) scans were acquired under bolus-free and bolus-applied conditions after delineating and marking the scalp boundary with CT-SPOTS line #118 (FLAIR, Tokyo, Japan). For the bolus-applied condition, CT imaging was performed using two different bolus placement patterns, as described below. All CT images were obtained using a SOMATOM go. Top scanner (Siemens Healthineers, Erlangen, Germany) with a slice thickness of 1 mm, covering the region from the head to the neck. Using the scalp markers as a reference, a virtual clinical target volume (CTV) was defined as the subcutaneous region extending from 5 mm beneath the skin surface to the outer surface of the skull, excluding the cranial bone. The anterior border of the CTV was defined to approximately correspond to the hair-bearing region of the scalp, reflecting the extent of target volumes encountered in our clinical experience with scalp angiosarcoma. A 5 mm margin was added to generate the planning target volume (PTV), with the external margin cropped to the scalp surface in accordance with the method described by Inui *et al*. [12]. Organs at risk (OARs) included brain, optic nerves and lenses. To avoid interobserver variability, all contours were generated by a single radiation oncologist. Treatment planning was performed using Eclipse version 15.6 (Varian Medical Systems, Palo Alto, CA, USA).

A 1 cm–thick tissue-equivalent bolus (Bolx-I 31102; CQ Medical, Orange City, IA, USA) was employed. This bolus is available in two configurations: a reusable cover type and a nonreusable coverless-type with higher conformity. Following preliminary testing, the cover type was chosen because it was more suitable for application over large and anatomically complex curved surfaces. Notably, the cut edges of the cover-type bolus exhibited strong adhesiveness, and intentional alignment of these edges at junction sites enhanced setup reproducibility. Consequently, the cover-type bolus was adopted in this study.

After evaluating bolus application strategies in terms of geometric precision, reproducibility and setup efficiency, a three-segment bolus application approach, termed the patch bolus method, was developed (Fig. 1). One bolus segment was placed from the frontal to the occipital region (Fig. 1A), while the remaining two segments were placed over the bilateral temporal regions (Fig. 1B). Finally, the patient was immobilized using a thermoplastic mask applied over the bolus (Fig. 1C). The occipital segment was deliberately cut slightly larger than the headrest to improve placement stability. Representative examples of the fabricated bolus segments are shown in Fig. 2. Although the target volume encompassed the entire scalp, it could be adequately covered using a single 30 cm × 30 cm × 1 cm sheet. The bolus applied to the bilateral temporal regions was initially cut into an oversized shape and subsequently trimmed in a stepwise manner to conform optimally to the target anatomy. Junctions between bolus segments were primarily maintained using the intrinsic adhesiveness of the bolus material and were further secured with adhesive tape. Because highly adhesive bolus materials are challenging to shape, multiple cutting tools were assessed. A large-diameter (60 mm) round-blade rotary cutter (Rotary Cutter LL type 136B; OLFA, Osaka, Japan) allowed efficient and precise cutting of the bolus material, enabling practical implementation of the patch bolus method with acceptable reproducibility.

**Fig. 1:**
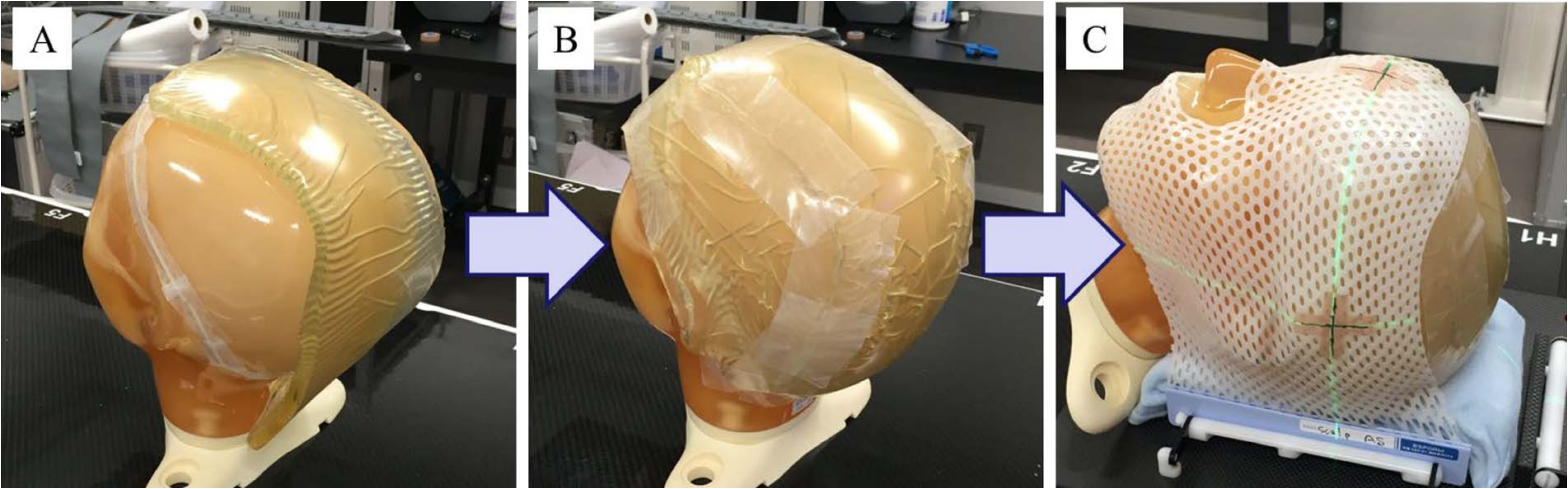
Bolus application using the patch bolus method. The bolus is applied from the frontal to the occipital region, followed by placement over the bilateral temporal regions, and is secured with a thermoplastic mask.

**Fig. 2:**
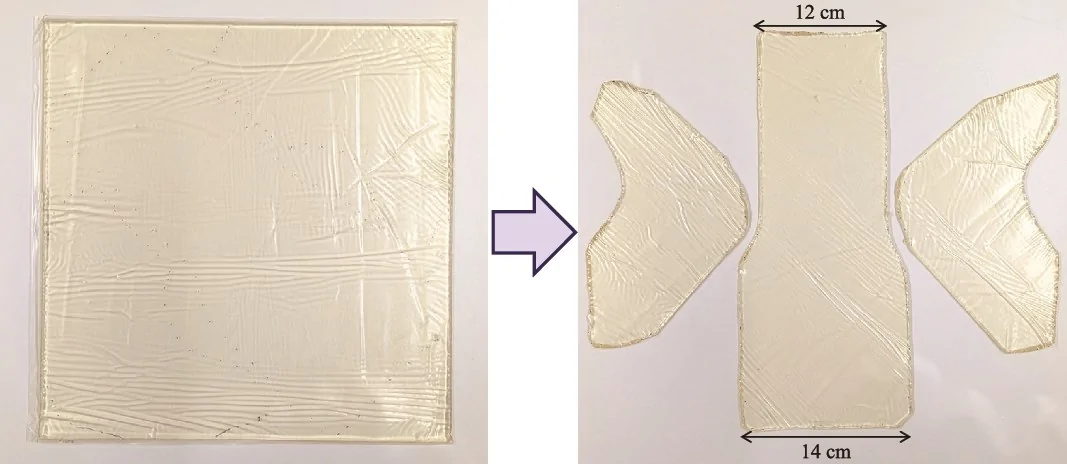
Representative samples of the unmodified 30 cm × 30 cm × 1 cm bolus (left) and the corresponding bolus segmented into three sections (right).

### Treatment planning

Advanced photon techniques such as helical tomotherapy and HyperArc have been reported for this indication [11–13], with evidence suggesting superior performance of the latter [12]. In the present study, HyperArc was selected because of its potential to achieve the highest degree of dose conformity among photon-based approaches. By employing a single isocenter in combination with multiple automatically optimized noncoplanar arcs, HyperArc enables highly conformal dose distributions with steep dose gradients. In addition, its automated workflow facilitates efficient treatment delivery within a relatively short time frame. Originally developed as a highly precise radiotherapy technique for patients with multiple brain metastases [14], it has recently begun to be explored for application in the treatment of head and neck cancers. HyperArc treatment plans were generated in accordance with the methodology described by Inui *et al*. [12], using four half-field noncoplanar arcs with 6 MV flattening filter-free photon beams. All fields were closed at the X1 jaw and opened at the X2 jaw to achieve tangential irradiation of the scalp while limiting dose to the brain. During optimization, the jaw-tracking function was enabled. In addition, the SRS NTO function was applied to promote steep spatial dose fall-off from target-specific dose levels to low asymptotic dose levels. All plans were created for a TrueBeam STx (Varian Medical Systems) equipped with high-definition multi-leaf collimator, with leaf widths of 2.5 mm for the central 32 leaves and 5 mm for the remaining leaves. The prescription dose was 70 Gy, defined as the mean dose to the PTV. Optimization objectives were established based on the dosimetric criteria reported by Inui *et al*. [12, 13]. Specifically, the PTV D_99%_ was required to exceed 63 Gy, while the dose to 0.1 cc (*D*_0.1cc_) of the PTV was further constrained to no >110% of the prescription dose. Brain dose was minimized to the extent reasonably achievable. Dose calculations were performed using the Acuros XB algorithm with a calculation grid size of 2 mm.

### Analysis

#### Comparison of virtual bolus, single-sheet bolus and patch bolus methods

Wrinkles and small air gaps were observed on CT images when the cover-type bolus was applied. To establish an idealized reference condition without air gaps, a 1 cm–thick virtual bolus was created within the treatment planning system. In addition, a single-sheet bolus method was evaluated, in which a 30 cm × 30 cm × 1 cm bolus sheet was applied to the scalp as a single unsegmented piece without cutting. To compare the conformity of the patch bolus and single-sheet bolus methods, air gaps between the bolus and scalp surface were quantitatively evaluated. Previous studies have assessed bolus conformity using either distance-based parameters or air-gap volume [15, 16]. However, because the single-sheet bolus method may produce highly irregular and spatially heterogeneous bolus–scalp interfaces over the extensive scalp surface, a single distance measurement may not adequately characterize the overall extent of bolus–scalp separation. Therefore, air-gap volume was selected as the primary metric for conformity evaluation in the present study. Air cavities were automatically segmented in the Eclipse using a CT-number threshold ranging from −1000 to −800 HU. A structure representing the potential air-gap region was then generated by subtracting the head structure from the external body contour including the bolus. The final air-gap structure was obtained using a Boolean AND operation between the segmented air cavities and the generated air-gap region. The resulting structure volume (cc) was recorded as the air-gap volume for each bolus method. Following the air-gap analysis, treatment plans for all three conditions were generated using identical dose constraints and optimization parameters. Dose distributions and dose–volume histogram (DVH) metrics were then compared across the three bolus configurations.

#### Impact of bolus reproducibility on dose distribution

In clinical practice, the treatment planning CT scan is typically acquired with the physical bolus in place to meet immobilization requirements. Accordingly, the treatment plan created on the initial bolus-applied CT was designated as the reference plan (Ref-plan). The bolus was subsequently reapplied to the phantom using an identical procedure, and CT acquisition was repeated 10 times. To simulate routine clinical practice, all bolus applications were performed by the same two operators throughout the study. Because the single-sheet bolus method demonstrated extremely poor reproducibility and rendered thermoplastic mask fitting impractical, only the patch bolus method was included in this analysis.

The Ref-plan was copied to each CT dataset, followed by dose recalculation. To exclude setup-related variability associated with repeated scans, all CT datasets were rigidly registered and resampled to the reference CT using 3D Slicer (version 5.0.2; Brigham and Women’s Hospital, Boston, MA, USA). This process ensured that any differences in dose distribution reflected variations in bolus application alone. Using the Ref-plan as the baseline, dose variations to the CTV and OARs across the 10 datasets were analyzed using DVH metrics.

## RESULTS

Figure 3 presents axial images with the virtual bolus, single-sheet bolus and patch bolus methods. Marked bilateral air gaps were evident with the single-sheet bolus method, whereas air gaps were substantially reduced when the patch bolus method was used. Consistent with the visual findings, the measured air-gap volume decreased from 36.3 cc with the single-sheet bolus to 10.9 cc with the patch bolus, corresponding to a reduction of >70%. Dose distributions and DVHs for the three planning strategies are shown in Figs 4 and 5, respectively. Although the patch bolus method demonstrated localized dose variations attributable to minor differences in bolus thickness or small residual air gaps, no clinically relevant hot or cold spots were observed within the target volume. In contrast, despite the absence of obvious abnormalities on visual inspection of the dose distribution, the single-sheet bolus method resulted in inferior PTV coverage, particularly in regions with inadequate bolus contact. This observation was quantitatively confirmed by the PTV D_99%_ values, which were 64.1, 62.1 and 63.8 Gy for the virtual bolus, single-sheet bolus and patch bolus methods, respectively. Notably, only the single-sheet bolus method failed to achieve the planning objective of PTV *D*_99%_ > 63 Gy. Figure 6 displays central axial slices from all 11 datasets acquired using the patch bolus method. Table 1 summarizes both the absolute dosimetric values and the corresponding relative variations from the Ref-plan obtained, following 10 repeated cycles of patch bolus removal and repositioning. The observed variations were small, with all evaluated parameters demonstrating narrow ranges across repeated measurements. Figure 7 illustrates relative deviations in all evaluated parameters from the Ref-plan. With the exception of the left lens, variations in all evaluated parameters were within 2%. Although variations exceeding 5% were observed for the left lens, the corresponding absolute dose values remained low.

**Fig. 3:**
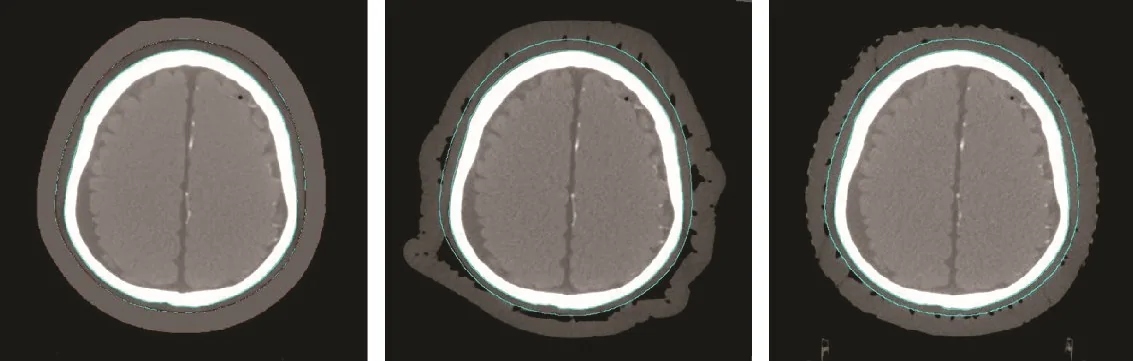
Axial views of the virtual bolus method (left), single-sheet bolus method (middle) and patch bolus method (right). The contour indicates the clinical target volume.

**Fig. 4:**
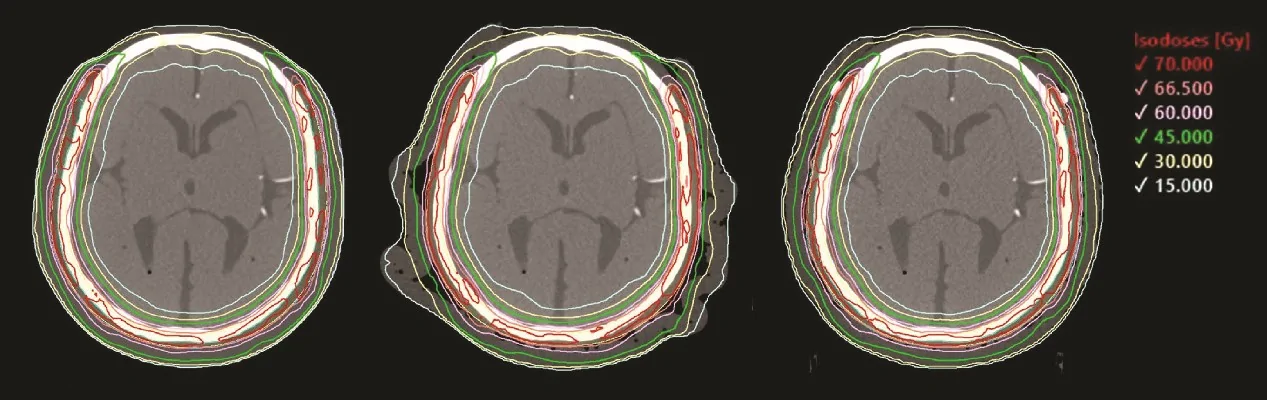
Dose distributions for the virtual bolus method (left), single-sheet bolus method (middle) and patch bolus method (right).

**Fig. 5:**
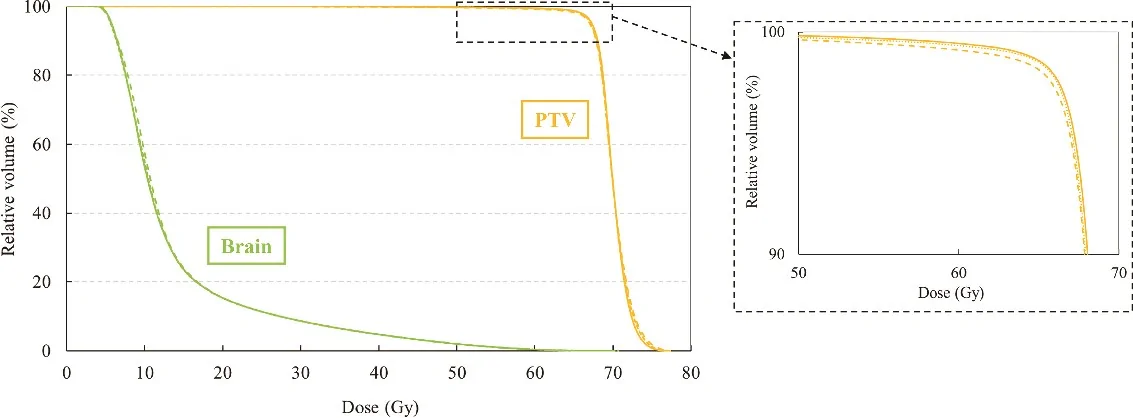
Dose–volume histograms for the planning target volume and the brain. Solid, dashed and dotted lines represent the virtual bolus method, single-sheet bolus method and patch bolus method, respectively. The right panel shows an enlarged view of the shoulder region of the planning target volume dose–volume histogram.

**Fig. 6:**
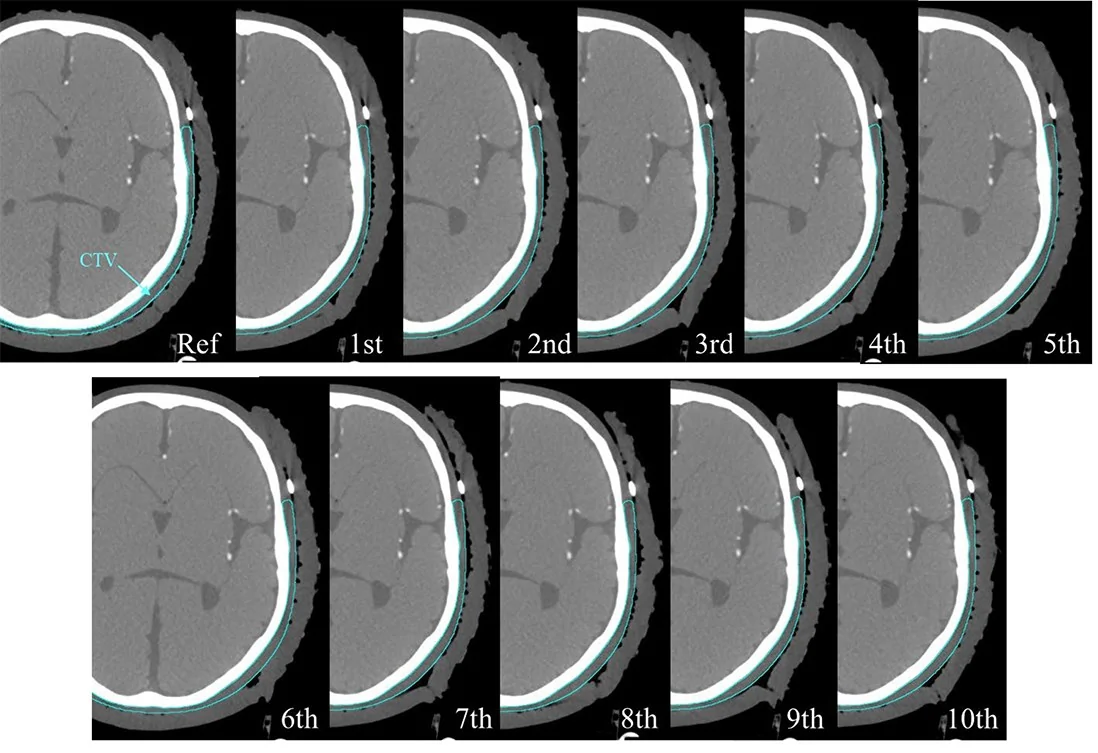
Central axial slices from all 11 datasets acquired using the patch bolus method.

**Table 1:** Absolute dosimetric values and relative variations from the reference plan for 10 repeated patch bolus applications

Dosimetric parameters	Absolute dose (Gy) Mean ± SD (range)	Relative variations (%) Mean ± SD (range)
CTV *D*_98%_	67.45 ± 0.09 (67.27–67.55)	−0.26 ± 0.13 (−0.52 to −0.10)
CTV *D*_0.1cc_	76.67 ± 0.56 (75.73–77.34)	1.21 ± 0.74 (−0.03 to −2.10)
Brain *D*_mean_	14.59 ± 0.02 (14.56–14.62)	0.03 ± 0.14 (−0.21to 0.21)
Rt optic nerve *D*_max_	13.66 ± 0.06 (13.56–13.72)	0.42 ± 0.43 (−0.29 to 0.88)
Lt optic nerve *D*_max_	12.22 ± 0.03 (12.18–12.26)	0.58 ± 0.21 (0.25–0.91)
Rt lens *D*_max_	8.04 ± 0.06 (7.99–8.13)	0.94 ± 0.47 (0.56–1.68)
Lt lens *D*_max_	7.79 ± 0.20 (7.55–8.12)	0.92 ± 2.63 (−2.20 to 5.18)

Abbreviations: Rt, right; Lt, left; SD, standard deviation.

**Fig. 7:**
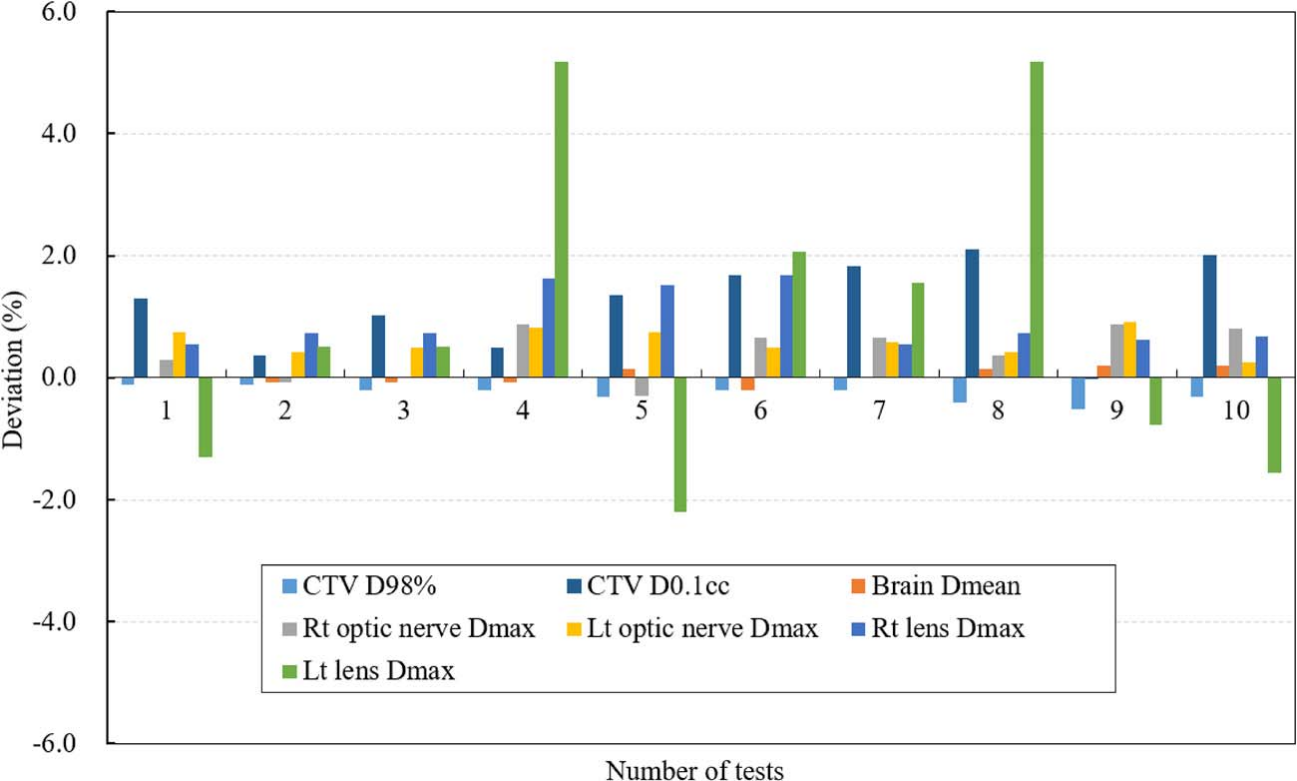
Relative variations in clinical target volume dose metrics (*D*_98%_ and *D*_0.1cc_), mean brain dose and maximum doses to the bilateral optic nerves and lenses across the 10 recalculated treatment plans compared to the reference plan.

## DISCUSSION

Accurate and reproducible bolus placement over large, highly curved surfaces such as the scalp has long been recognized as a major technical challenge in TSI. To address this issue, various alternative approaches have been proposed, including the use of scuba caps, thermoplastic sheets and patient-specific boluses fabricated using 3D printing [17–20]. Although these approaches can improve bolus conformity, they often increase fabrication complexity and impose additional burdens on clinical workflow. More recently, helmet-shaped boluses assembled by cutting and joining flat commercial bolus sheets have been reported [21]. However, these techniques reportedly require >30 minutes to construct and may still produce substantial air gaps, even under phantom conditions. When irradiation of the entire scalp is required, segmentation and assembly of the bolus are essentially unavoidable. Nevertheless, the high adhesive properties of commercially available bolus materials have traditionally limited their practical modification. In the present study, this limitation was overcome through the use of a large-diameter rotary cutter, enabling the development of the patch bolus method as a clinically feasible technique. By leveraging the intrinsic adhesiveness of the cut bolus surfaces, stable fixation at segment junctions was achieved. Although minor wrinkles and small residual air gaps could not be completely eliminated, comparison with the virtual bolus condition demonstrated that these imperfections were unlikely to result in clinically meaningful dose degradation. While the method itself is not technologically novel, its simplicity and ease of implementation allow it to provide practical value in routine clinical settings comparable to that of more sophisticated approaches.

Repeated bolus application with serial CT acquisitions showed that variations exceeding 5% were observed only for the left lens dose. Although the right lens demonstrated slightly greater variability than other evaluated metrics, these variations remained within a narrow range. Importantly, both lenses were located at a sufficient distance from the CTV, and the corresponding absolute dose levels were low. Given the small volume of the lens and the presence of steep internal dose gradients, small absolute dose differences may be magnified when expressed as relative changes. In addition, minor variations in bolus placement may have contributed to the observed asymmetry between the right and left lens doses. Because the magnitude of the variation was small, the relative contributions of bolus placement variability and beam geometry could not be clearly distinguished. Taken together, the observed variability likely overestimates the true clinical impact, and the patch bolus method can be considered dosimetrically acceptable with respect to dose stability for OARs.

Several previous studies have proposed alternative approaches to improve bolus conformity, including rigid or patient-specific boluses [17–20]. However, TSI typically involves high prescription doses, and acute skin toxicities, such as dermatitis or bleeding, are not uncommon during treatment. These clinical scenarios often require flexible, day-to-day adjustments in bolus placement, which rigid or highly customized devices may not readily accommodate. In contrast, the patch bolus method permits real-time visual assessment of scalp conditions and flexible modification during application. In addition to its practical simplicity, the use of commercially available bolus materials may also offer potential cost advantages compared with customized bolus fabrication approaches. Notably, the proposed technique reduces bolus application time to ~5 minutes while limiting air gaps to a minimal extent, representing a substantial improvement over previously reported flat bolus methods from the perspective of clinical workflow [21].

Several limitations of this study should be acknowledged. First, although the adhesive properties at bolus junctions contributed to improved fixation and reproducibility, repeated use may lead to gradual degradation of adhesiveness. However, based on our experience with storing and managing cut bolus materials for >1 year, no notable deterioration was observed, suggesting minimal impact on durability. Second, the PTV was cropped at the external body surface. This condition was intentionally adopted to enable direct comparison with the single-sheet bolus method, in which large air gaps were expected. However, such external cropping is suboptimal for routine clinical practice because it reduces robustness to setup uncertainties [22]. Given that the patch bolus method markedly limits the impact of air gaps, extending the PTV margin beyond the body surface in actual clinical settings would likely enhance robustness against setup errors. Third, the extensive use of noncoplanar beam arrangements in HyperArc may have attenuated the dosimetric impact of bolus displacement. Ostheimer *et al*. reported that noncoplanar IMRT exhibits dosimetric characteristics distinct from those of coplanar IMRT [9]. Therefore, the robustness observed in this study may be partially attributable to the noncoplanar geometry intrinsic to HyperArc. Importantly, the patch bolus method itself is not restricted to HyperArc and may also be applicable to conventional VMAT, IMRT and even electron beam therapy. Fourth, this study was conducted using a single anthropomorphic head phantom and therefore did not account for interpatient variations in head size, scalp curvature, anatomical contour or other patient-specific factors that may influence bolus fitting and junction uniformity. Although the patch bolus method demonstrated favorable conformity and reproducibility under the present experimental conditions, its performance in actual clinical patients with diverse anatomical characteristics remains to be validated. Future clinical studies involving a broader range of patient anatomies are warranted to confirm the reproducibility, robustness and generalizability of the proposed approach. Because the required level of geometric and dosimetric precision differs among irradiation techniques, institution-specific validation is recommended before clinical implementation of the patch bolus method.

## CONCLUSION

This study investigated practical strategies to improve the placement accuracy of commercially available bolus and experimentally evaluated the dosimetric impact of bolus reproducibility in VMAT-based TSI. Although application of a commercial bolus over the entire scalp inevitably involves some degree of uncertainty because of the large and highly curved surface geometry, the results demonstrate that clinically acceptable treatment quality can be achieved through appropriate technical modifications.

Dosimetric analysis showed that HyperArc is relatively robust to localized variations in bolus placement, with minimal effects on target coverage and doses to OARs. The proposed patch bolus method improved bolus conformity while maintaining flexibility during application and provided adequate reproducibility and dosimetric stability. From a clinical perspective, this technique represents a feasible and adaptable approach for the routine implementation of TSI using external beam radiotherapy.

## PRESENTATION AT A CONFERENCE

We presented at the 38th JASTRO annual meeting.
